# Non-blood circulating tumor DNA detection in cancer

**DOI:** 10.18632/oncotarget.19942

**Published:** 2017-08-04

**Authors:** Muyun Peng, Chen Chen, Alicia Hulbert, Malcolm V. Brock, Fenglei Yu

**Affiliations:** ^1^ Department of Thoracic Surgery, The Second Xiangya Hospital, Central South University, Changsha, Hunan, P.R China; ^2^ Department of Surgery, The Johns Hopkins University School of Medicine, Baltimore, Maryland, USA

**Keywords:** liquid biopsy, cell free DNA, early diagnosis, cancer

## Abstract

Tumor DNA contains specific somatic alterations that are crucial for the diagnosis and treatment of cancer. Due to the spatial and temporal intra-tumor heterogeneity, multi-sampling is needed to adequately characterize the somatic alterations. Tissue biopsy, however, is limited by the restricted access to sample and the challenges to recapitulate the tumor clonal diversity. Non-blood circulating tumor DNA are tumor DNA fragments presents in non-blood body fluids, such as urine, saliva, sputum, stool, pleural fluid, and cerebrospinal fluid (CSF). Recent studies have demonstrated the presence of tumor DNA in these non-blood body fluids and their application to the diagnosis, screening, and monitoring of cancers. Non-blood circulating tumor DNA has an enormous potential for large-scale screening of local neoplasms because of its non-invasive nature, close proximity to the tumors, easiness and it is an economically viable option. It permits longitudinal assessments and allows sequential monitoring of response and progression. Enrichment of tumor DNA of local cancers in non-blood body fluids may help to archive a higher sensitivity than in plasma ctDNA. The direct contact of cancerous cells and body fluid may facilitate the detection of tumor DNA. Furthermore, normal DNA always dilutes the plasma ctDNA, which may be aggravated by inflammation and injury when very high amounts of normal DNA are released into the circulation. Altogether, our review indicate that non-blood circulating tumor DNA presents an option where the disease can be tracked in a simple and less-invasive manner, allowing for serial sampling informing of the tumor heterogeneity and response to treatment.

## INTRODUCTION

Gene mutation and methylation play essential roles in tumorigenesis and metastasis [1–3]. Tumor DNA contains specific somatic alterations that are crucial for the diagnosis and treatment of cancer. Due to the spatial and temporal intra-tumor heterogeneity, multi-sampling is needed to adequately characterize the somatic alterations [4–6]. Tissue biopsy, primarily the “gold standard” for diagnosis, however, is limited by the restricted access to sample and the challenges to recapitulate the tumor clonal diversity [7, 8]. Plasma circulating tumor DNA (ctDNA) is DNA fragments that contain the tumor-specific somatic alterations in the blood. Recent studies have demonstrated the application of plasma ctDNA detection in the tumor diagnosis and monitoring [9–11]. It is of non-invasive and can be collected repeatedly with minimal discomfort to the patient. It also reflects the total tumor burden and genetic heterogeneity.

**Figure 1:**
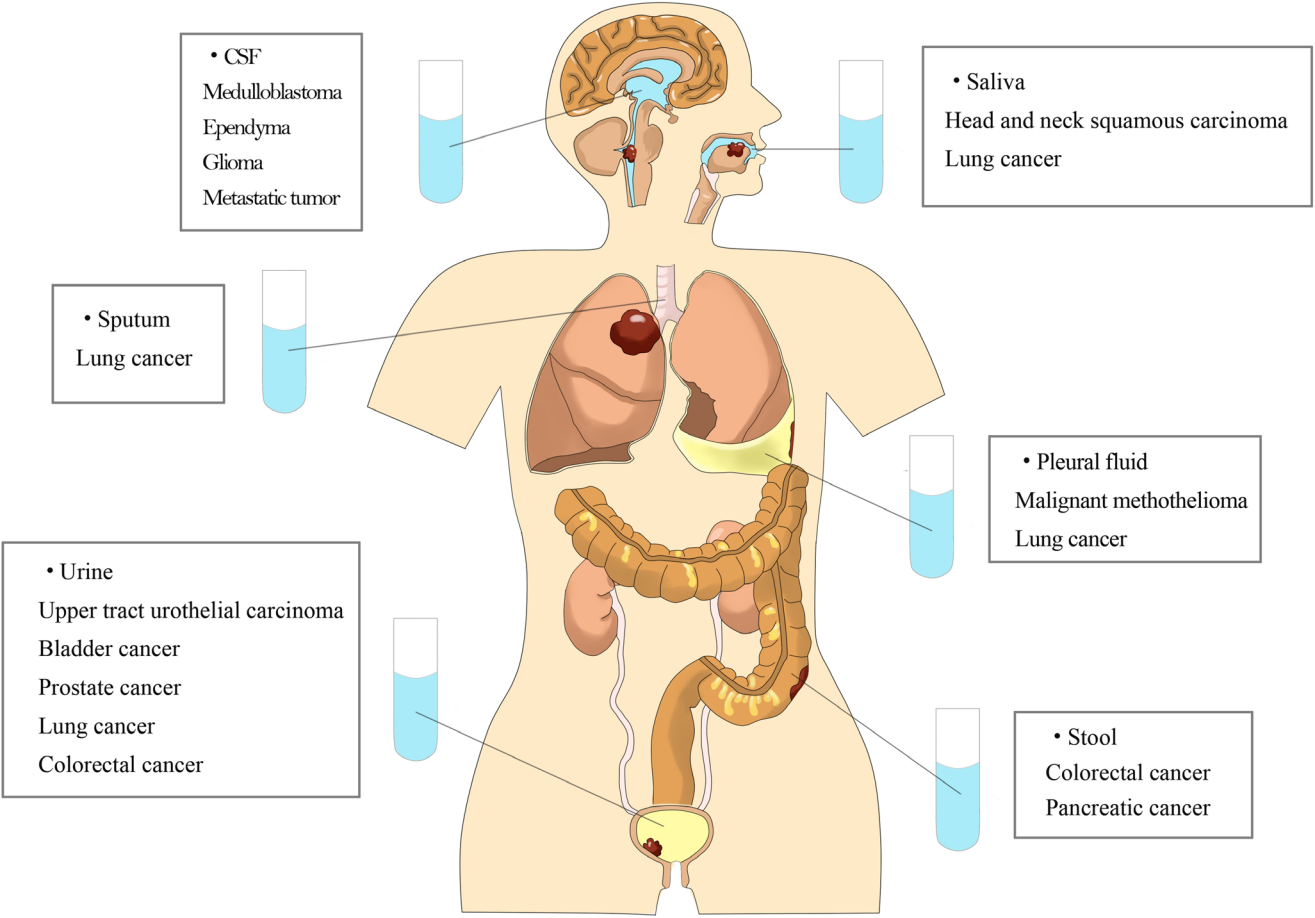
Tumor DNA can be detected in various kinds of non-blood body fluids

Non-blood circulating tumor DNA are tumor DNA fragments presents in other body fluids, such as urine, saliva, sputum, stool, pleural fluid, and cerebrospinal fluid(CSF). Recent studies have demonstrated the presence of tumor DNA in these non-blood body fluids and their application to the diagnosis, screening, and monitoring of cancers [12] (Figure 1). The collection of these body fluids, such as urine, saliva, sputum, and stool, are relatively safe, non-invasive, economic and can be performed at home, without professional help [13, 14]. It also applies perfectly to patients with anemia, which is quite common in advanced stage cancer patients. Moreover, enrichment of tumor DNA of local cancers in non-blood body fluids may help to archive a higher sensitivity than in plasma. Non-blood body fluids are a viable alternative to blood samples as a source of DNA for tumor diagnosis and monitoring.

### Sources of non-blood tumor DNA

**Figure 2:**
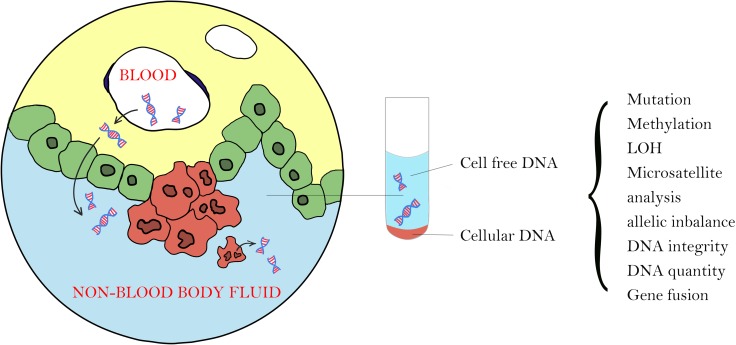
There are 2 types of tumor DNA in non-blood body fluid: cellular tumor DNA from local tumor cells that shed into the body fluid and cell-free tumor DNA from plasma cell-free DNA or from local tumor cells due to necrosis or apoptosis

There are 2 types of non-blood tumor DNA: genomic DNA from local tumor cells that shed into the body fluid (cellular tumor DNA) and cell-free tumor DNA (cfDNA) from plasma cell-free DNA or from neighboring tumor cells due to necrosis or apoptosis (Figure 2).

Urinary cfDNA mainly originates from plasma cfDNA that pass the kidney barrier [15]. Circulating tumor cfDNA is highly fragmented and primarily present in the blood as part of super molecule complexes, such as nucleosomes. During circulation, cfDNA was filtrated from the blood into the primary urine through the kidney barrier, which has been proved to be permeable to DNA molecules [16]. However, it is not known of the mechanism of DNA translocation from the bloodstream into the urine. Limited by the basal membrane and slit membranes between podocytes pedicles, only complexes smaller than 6.4 nm in diameter and with a molecular weight no greater than 70 kDa can pass through the kidney barrier and enter the nephron. It corresponds to DNA of about 100 bp in size, which is smaller than mononucleosome [15]. Moreover, the negatively charged cfDNA might face an additional barrier because of the negative charge of the glomerular basement membrane. It might be due to the non-globular shape or by the deformability of the DNA complexes. Another explanation is that cfDNA may be covered by liposomes, which make their penetration through the kidney barrier theoretically possible [17, 18]. Besides, renal permeability might increase for some physical and pathological conditions, such as pregnancy, cancer, and inflammation.

Ying *et al*. reported two size categories of urinary DNA: low molecular weight (MW) urine DNA and high MW urine DNA. Low MW class of urine DNA is between 150 to 250 bp and derived from the circulation, while the high MW urine DNA is greater than 1 kb and mostly from the cells shed into the urinary tract [13]. Similar findings were also reported in CSF. Size distribution of CSF cfDNA peaks at 160 and 340bp indicates an apoptotic source. CSF cellular DNA is of larger size and may origin from blood cells and tumor cells in CSF [19]. Saliva DNA can also be locally generated by cell necrosis, apoptosis, and exfoliation, or by active transport, passive diffusion or ultrafiltration from the plasma cfDNA [20]. The possible origins of pleural DNA also include ultrafiltration from the plasma (cfDNA) and local dying or apoptotic cells (cellular DNA) [21].

### Non-blood tumor DNA and cancer

#### Urinary tumor DNA

Urinary cellular DNA origins from urological cancer cells shed into urine [22, 23]. In 1991, Sidransky *et al*. first identified p53 gene mutations in the urine sediment of bladder cancer patients [24]. In 2001, Carsten *et al*. demonstrated the presence of GSTP1 promoter hypermethylation in the urine sediments of prostate cancer patients [25]. Since then, gene mutations such as p53 [26], TERT [27, 28], FGFR3 [29, 30] mutations and gene hypermethylation changes such as GSTP1 [31], RARβ [31, 32] were reported in the urine of urothelial carcinomas patients (Table 1). In a 2-year follow-up of patients with superficial bladder transitional cell carcinoma after transurethral resection, Camille *and colleagues* assessed the diagnostic and prognostic performance of urinary cellular FGFR3 mutation analysis. Urinary cellular FGFR3 mutation has a sensitivity of 73% (95% CI, 0.58–0.89) and a specificity of 87% (95% CI, 0.82–0.93) in the diagnosis of cancer recurrence after transurethral resection, and a sensitivity of 70% (95% CI, 0.54–0.86) and specificity of 87% (95% CI, 0.76–0.98) in the prediction of recurrence within 2 years after surgery [38].

**Table 1:** Urinary tumor DNA detection in cancer

Tumor	Sample	Author	Type	Gene	Method	Patients/ Control	Sensitivity/ Specificity
UTUC	cellular	Monteiro *et al*. [33]	Methylation	GDF15/TMEFF2/VIM	QMSP	22/20	91%/100%
PC	cellular	Noel *et al*. [26]	Mutation	TP53/FGFR3	FASAY and SNaPshot system	103/NA	46%/81%
PC	cellular	Minciu *et al*. [34]	Methylation	GSTP1	MSP	31/34	98%/87%
PC	cellular	Daniunaite *et al*. [31]	Methylation	RASSF1/RARB/GSTP1	Real time-MSP	34/ NA	82%/NA
PC	cfDNA	Salvi *et al*. [35]	DNA Integrity	c-MYC/HER2/AR	Real time-PCR	67/64	58%/44%
PC	cfDNA	Casadio *et al*. [36]	DNA Integrity	c-Myc/BCAS1/HER2	Real time-PCR	29/25	79%/84%
PC	cellular	Zhu *et al*. [37]	Gene fusion	TTTY15-USP9Y	Real time-PCR	75/151	84%/77.5%
BC	cellular	Couffignal *et al*. [38]	Mutation	FGFR3	allele-specific PCR	191/ NA	73%/87% (recurrence)
BC	cellular	Chihara *et al*. [39]	Methylation	SOX1/TJP2/MYOD/HOXA9VAMP8/CASP8/SPP1/IFNG/CAPG/HLADPA1/RIPK3	Pyrosequencing	73/18	100%/100%
BC	cellular	Beukers *et al*. [40]	Methylation	OSR1/SIM2/OTX1/MEIS1/ONECUT2	bisulfite-specific PCR	54/115	82%/82%
BC	cellular	Kandimalla *et al*. [41]	Methylation	OTX1/ONECUT2/OSR1	quantitative assessment	101/70	68%/90% (recurrence)
BC	cellular	Baquero *et al*. [42]	Methylation	18 tumor suppressor genes	MS-MLPA	100/28	7–42%/ 64.3–92.9%
BC	cellular	Scher *et al*. [43]	Methylation	BCL2/CDKN2A/NID2	Nested MSP	42/21	81%/86%
BC	cellular	Reinert *et al*. [44]	Methylation	EOMES/HOXA9/POU4F2/TWIST1/VIM/ZNF154	MethyLight	184/35	88–94%/43–67%
BC	cellular	Berrada *et al*. [45]	Methylation	APC/RARβ/survivin	MSP	32/NA	93.7%/NA
BC	cellular	Eissa *et al*. [32]	Methylation	RARβ(2)/APC	MSP	210/110	87.3%/97.6%
BC	cellular	Chung *et al*. [46]	Methylation	MYO3A/CA10/SOX11/ NKX6-2/PENK/DBC1	QMSP	128/110	81–85%/95–97%
BC	cellular	Costa *et al*. [47]	Methylation	GDF15/TMEFF2/VIM	Real time-QMSP	51/59	94%/90%
BC	Total DNA	Karnes *et al*. [48]	Mutation Methylation	Mutation: FGFR3 Hypermethylation: TWIST1/NID2*	Real time-PCR and MSP	58/690	87.9%/56.2% (recurrence)
BC	Total DNA	Shore *et al*. [30]	Mutation Methylation	Mutation: FGFR3 Hypermethylation: NID2/VIM*	PCR-clamping and MSP	63/670	90.5%/34.5% (recurrence)
BC	cellular	Dahmcke *et al*. [49]	Mutation methylation	Mutation: TERT/FGFR3 Methylation: SALL3/ONECUT2/CCNA1/BCL2/EOMES/VIM	ddPCR and MethyLight	99/376	97.0%/76.9%
BC	cfDNA	Brisuda *et al*. [50]	Quantity	-	Real time-PCR	66/34	42.4%/91.2%
BC	cfDNA	Casadio *et al*. [51]	DNA integrity	c-Myc/BCAS1/HER2	Real time-PCR	51/46(BUD),32(HI)	73%/83%(BUD),84%(HI)
BC	cellular	van Tilborg *et al*. [52]	AI, LOH	12 microsatellites markers	MA	102/NA	58%/NA
BC	cellular	Traczyk *et al*. [53]	LOH	TP53/RB1/CDKN2A/ARF	PCR	125/NA	34.3%/NA
NSCLC	cfDNA	Reckamp *et al*. [54]	Mutation	EGFR	NGS	60/ NA	T790M: 72%/96% L858R: 75%/100% Exon 19 Del: 67%/94%
NSCLC	cfDNA	Chen *et al*. [55]	Mutation	EGFR	ddPCR	150/NA	88%/NA
CRC	Total DNA	Song *et al*. [56]	Methylation	VIM	MethyLight	20/NA	75%/NA
CRC	Total DNA	Su *et al*. [13]	Mutation	p53	Restriction-Enriched PCR	20/NA	83%/NA

BC = bladder cancer; BUD = benign urogenital diseases; CRC = colorectal cancer; HI = healthy individuals; NSCLC = non-small cell lung cancer; PC = prostate cancer; UTUC = upper tract urothelial carcinoma; ddPCR = droplet digital polymerase chain reaction; MSP = methylation-specific PCR; NGS = next generation sequencing; QMSP = quantitative MSP; FASAY = functional analysis of separated allele in yeast; MS-MLPA = methylation-specific multiplex ligation-dependent probe amplification; MA = microsatellite analysis; AI = allelic imbalance; LOH = loss-of-heterozygosity; NA = not available.

*Panel includs a protein biomarker matrix metalloproteinase(MMP)-2.

Urinary cfDNA can be detected in non-urothelial carcinoma patients. The p53 mutation was found by Selena *et al*. in the urine of hepatocellular carcinoma patients and most of the p53 mutation was detected in the low MW urine DNA fraction [57]. Chen *et al*. detected EGFR mutations in urinary cfDNA in non-small cell lung cancer (NSCLC) patients with a concordance of 88% to their primary tumors [55].

Su *et al*. demonstrated that DNA methylation in urine is better in predicting recurrence than cytology and cystoscopy in bladder cancer patients after transurethral resection. By using a three-marker panel (SOX1, IRAK3, and L1-MET), they could predict tumor recurrence in 80% of patients, which is superior to cytology (35%) and cystoscopy (15%) [58]. Reckamp *et al*. detected the EGFR activating mutations and the T790M resistance mutation in urine and plasma of NSCLC patients by short footprint mutation enrichment next generation sequencing assays. They found that with a recommended specimen volume (90–100 mL), the sensitivity of urine and plasma are comparable [54]. Chen *et al*. conducted a serial monitoring trial to detect urinary cfDNA of EFGR mutation in NSCLC patients receiving EGFR-TKIs. They found a concordance in the quantity of urinary cfDNA and plasma cfDNA at baseline. During the treatment, a concordance in the decline of the quantity of cfDNA was also observed. Nevertheless, a more significant decrease in urinary cfDNA than plasma cfDNA was found during the early phase of monitoring as a result of treatment, which indicated that urinary cfDNA might potentially be of higher sensitivity [55].

#### Saliva tumor DNA

Saliva provides good-quality genomic DNA, which is comparable to blood as a template for genotyping [59, 60]. Salivary DNA has been used in germline mutations detection for various cancer screening, such as breast cancer [61] and braintumor [62].

In 2000, Liao et al. detected p53 gene mutation in the saliva of oral squamous cell carcinomas patients [63]. In 2001, El-Naggar et al. performed microsatellite analysis at chromosomal regions frequently altered in head and neck squamous cell carcinoma (HNSCC) on matched saliva and tumor samples. Their results showed a statistically significant correlation in loss of heterozygosity (LOH) between saliva and tumor with some sets of markers [64]. Salivary DNA promoter hypermethylation analysis has also been found to be an efficient tool for diagnosis of HNSCC [65–67] (Table 2). In 2014, Wei et al. developed a novel core technology, called electric field-induced release and measurement (EFIRM) to detect EGFR mutations directly in body fluids with a multiplexable electrochemical sensor. They demonstrated that EFIRM could detect EGFR mutations in the saliva of NSCLC patients, with an area under the curve (AUC) of 0.94 in the detection of exon 19 deletion and an AUC of 0.90 in the detection of L858R mutation [75]. Using EFIRM, Pu et al. detected the EFGR exon 19 deletion and p.L858 mutations in saliva and plasma samples of 17 lung adenocarcinoma patients. They found a perfect concordance between saliva and tumor samples, with an AUC of 1.0 [14]. It is therefore suggested that there is a link between the peripheral circulatory system and the salivary glands that translocate cfDNA from the bloodstream into saliva [14].

**Table 2:** Saliva tumor DNA detection in HNSCC

Author	Type	Gene	Method	Patients/Controls	Sensitivity/Specificity
Sun *et al*. [68]	Methylation	TIMP3	QMSP	197/NA	NA/NA
Gaykalova *et al*. [69]	Methylation	ZNF14/ZNF160/ZND420	QMSP	59/ NA	57.6%/100%
Ovchinnikov *et al*. [70]	Methylation	MED15/PCQAP	MSP	46/49(5′-CpGs)44/45(3′-CpGs)	70%/63% (5′-CpGs)68%/58% (3′-CpGs)
Rettori *et al*. [71]	Methylation	CCNA1/DAPK/DCC/MGMT/TIMP3	QMSP	146/60	55%/76%
Demokan *et al*. [72]	Methylation	KIF1A/EDNRB	QMSP	71/61	77.4%/93.1%
Righini *et al*. [67]	Methylation	TIMP3/ECAD/p16^INK4a^/MGMT/DAPK/RASSF1	QMSP	60/ NA	78.3%/ NA
Ovchinnikov *et al*. [73]	Methylation	RASSF1A/DAPK1/ p16^INK4a^	MSP	143/31	80%/87%
Schussel *et al*. [74]	Methylation	EDNRB/DCC	QMSP	48/113	46%/72%

MSP = Methylation-Specific PCR; QMSP = Quantitative MSP; NA = not available.

#### Sputum tumor DNA

Sputum contains cells from the lungs and lower respiratory tract and provides sufficient tumor DNA for detection. Numerous studies have shown that sputum tumor DNA could be a promising tool for early detection of lung cancer (Table 3). In 1994, Mao *et al*. detected the K-ras mutation and p53 mutation in sputum samples of lung cancer patients. Using a PCR-based assay, they detected mutant DNA in the sputum of 8 of 10 patients with oncogene mutations in their primary tumor prior to clinical diagnosis [88]. DNA hypermethylation in sputum also helps the diagnosis of lung cancer [89]. Wang *et al*. carried out a meta-analysis to comprehensively review the evidence for using sputum aberrant methylation DNA to detect NSCLC. They found that the combined sensitivity was 62% (95% CI: 0.59–0.65), and specificity was 73% (95% CI: 0.70–0.75) [90]. Miglio *et al*. demonstrated that MGMT promoter methylation was present in small cell lung cancer and cytological samples were perfectly adequate for methylation analysis [91]. Flow cytometric DNA analysis of sputum cells also showed good sensitivity in the diagnosis of lung cancer. Compared with cytologic morphology of sputum cells, sensitivity of sputum DNA heteroploidy analysis was significantly higher (82.8% *vs* 27.6%, *P* < 0.005) [92].

**Table 3:** Sputum tumor DNA detection in lung cancer

Author	Type	Gene	Method	Patients/Control	Sensitivity/Specificity
Konno *et al*. [76]	Methylation	p16^INK4a^/APC/RARβ	MSP	78/95	78%/79%
Wang *et al*. [77]	Methylation	p16^INK4a^	PCR	34/21	32%/100%
Belinsky *et al*. [78]	Methylation	p16^INK4a^ /DAPK/H-cadherin/PAX5α/PAX5β/MGMT/RASSF1A	MSP	53/118	85%/35%
Olaussen *et al*. [79]	Methylation	HOX/p16^INK4a^/MAGE/MAGE	MSP	22/56	96%/79%
Cirincione *et al*. [80]	Methylation	RARβ2/p16^INK4a^/RASSF1A	MSP	18/112	50%/38%
Georgiou *et al*. [81]	Methylation	p16^INK4a^	MSP	80/40	69%/76%
Shivapurkar *et al*. [82]	Methylation	3-OST-2/RASSF1A/ p16^INK4a^ /APC	Quantitative Real time-PCR	13/23	62%/100%
van der Drift *et al*. [83]	Methylation	RASSF1A	RT-globin PCR	28/68	46%/99%
Hwang *et al*. [84]	Methylation	HOXA9	MSP	76/109	71%/55%
Zhang *et al*. [85]	Methylation	p16^INK4a^	MSP	44/20	61%
Destro *et al*. [86]	Mutation methylation	Mutation: K-ras Methylation: p16^INK4a^	PCR-RFLP and MSP	50/100	75%/96%
Wang *et al*. [87]	Methylation, LOH, MSI	methylation: p16^INK4a^/RARβ LOH: D9S286/D9S942/GATA49D12/D13S170 MSI: D9S942	MSP LOH analyses	79/22	81%/72%

MSP = Methylation-Specific PCR; PCR-RFLP = PCR Restriction Fragment Length Polymorphism; MSI = microsatellite instability; LOH = loss of heterozygosity.

DNA analysis from sputum was consistent with that from plasma in patients with lung cancer. By using a fluorescent PCR-based approach, Castagnaro *et al*. were able to assess the consistency of DNA microsatellite analysis of induced sputum. They demonstrated a significant trend in the percentage of the genetic alterations, found both in induced sputum and in blood samples, from healthy subjects to heavy smokers and lung cancer patients [93]. LOH and microsatellite instability (MSI) in at least one locus was observed in 55% of patients, in 18% of smokers, and in 4.5% of healthy subjects. These results showed that sputum DNA provided data that were consistent with those from plasma [93].

#### Stool tumor DNA

Stool is the fecal discharge from the bowels. It is biologically rational to use stool as a non-invasive sample for colorectal cancer (CRC). Stool DNA detection has a marked improvement of the sensitivity when compared to fecal blood tests. Compared to colonoscopy, which is currently the dominant screening test, stool DNA detection is patient-friendly and free from unpleasant cathartic bowel preparations and diet or medication restrictions. Furthermore, colonoscopy is operator dependent and has been shown to be not as effective detecting proximal lesions [94]. Stool DNA testing detects proximal and distal colorectal neoplasms equally well.

According to a meta-analysis involving 7524 patients, the pool sensitivities of stool DNA testing for CRC were 48% for single-gene and 77.8% for multiple-gene assays, and the specificities were 97% and 92.7%, respectively [95]. In a cross-sectional study including 9989 participants, Thomas *et al*. evaluated the effectiveness of multitarget stool DNA (mt-sDNA) test in colorectal-cancer screening. The mt-sDNA test included quantitative molecular assays for K-ras mutations, aberrant NDRG4, and BMP3 methylation, and β-actin, plus a hemoglobin immunoassay. The sensitivity for detecting colorectal cancer was significantly higher with mt-sDNA test than immunochemical test (FIT) (92.3% *vs*. 73.8%, *p* = 0.002) [96].

Besides CRC, stool tumor DNA can also be detected in other digestive system neoplasms. In 1994, Caldas demonstrated the presence of K-ras mutation in the stool of pancreatic cancer patients [97]. In a more recent work, Kisiel *et al* found a sensitivity of 67% and a specificity of 90% in detecting pancreatic cancer with a combination of stool mutated K-ras and methylated BMP3 detection [98].

Plasma based DNA tests, especially marking the aberrant methylation of SEPT9 gene, have been evaluated as a potential screening tool for CRC and advanced adenomas [99]. Ahlquist *et al*. conducted a case-control study to compare the sensitivities of multimarker stool DNA test and plasma methylated SEPT9 test in identifying patients with large adenomas or CRC. Their results demonstrated that mt-sDNA test had a significantly greater level of sensitivity than the plasma methylated SEPT9 test for detection of both CRC and large adenomas (87% *vs*. 60% CRC sensitivity and 82% *vs*. 14% adenoma sensitivity) [100]. The high sensitivity of stool DNA test may be related to disproportionately copious exfoliation of cancer cells, remarkably large functional surface area of neoplasms, enhanced survival of shed dysplastic cells, and relative stability and informativeness of tumor-associated DNA changes [100].

#### Pleural tumor DNA

Pleural effusions arise from a variety of systemic, inflammatory, infectious and malignant conditions. Malignant pleural effusion (MPE) is a devastating complication caused by a series of cancers, including lung cancer and mesotheliomas. Positive cytologic or tissue confirmation of malignant cells is necessary to establish a diagnosis. However, the sensitivity of pleural fluid cytological analysis is relatively low. Molecular biology techniques, such as analyses of DNA mutation and methylation status, have provided novel diagnostic tools for MPEs.

In 2006, Kimura *et al*. assessed the pleural effusion of 43 known NSCLC patients and found mutated EGFR in 11 of 43 cases [101]. Using peptide nucleic acid (PNA)-mediated real-time PCR clamping, Yeo *et al*. detected the EGFR mutation in pleural effusion of NSCLC patients with a sensitivity of 89% and a specificity of 100% [102]. Benlloch *et al*. examined the promoter methylation status of 4 genes (DAPK, RASSF1A, RARβ, p16^INK4a^) in patients with pleural effusion. Abnormal DNA methylation was detected in 58.5% of malignant pleural effusions, while in 0% of patients with benign pleural effusions [103]. Fujii *et al*. detected hypermethylated RASSF1A, p16^INK4a^, RARβ in both malignant pleural mesothelioma (MPM) and lung cancer. They found that the methylation ratios for the three genes were significantly higher in lung cancer than in MPM, which suggested that pleural fluid DNA could be a possible marker for differentiating MPM from lung cancer [104].

Using high resolution melting (HRM) analysis, Lin *et al*. assessed the pleural cfDNA and pleural cellular DNA of 13 known NSCLC cancer patients with EGFR mutation in matched biopsy tumor tissues, and found mutated EGFR in 12 and 9 of 13 cases, respectively [105]. Similar results were reported by Liu *et al*. using amplification refractory mutation system. Higher sensitivity of pleural cfDNA might be due to the tumor cells damaged under high speed of centrifugation, and DNA fragments were released from the nucleus, making up the dominant components of the supernatant [106].

#### CSF tumor DNA

Circulating tumor DNA has been detected in a variety of cancers. However, it is rarely found in patients with isolated brain tumors, presumably owing to the blood-brain barrier [107]. CSF is a clear, colorless body fluid that bathes the brain and spinal cord. It circulates nutrients and chemicals filtered from the blood and removes waste products from the brain. Examining the fluid can be useful in diagnosing many diseases of the nervous system, including brain tumors.

CSF tumor DNA provides a minimally invasive method to assess the genomic alterations of the tumor and monitor the therapy effect that helps both diagnosis and treatment. Using next generation sequencing approach, Pentsova *et al*. sequenced 341 cancer-associated genes in CSF of 53 patients with suspected or known CNS cancers. They detected high-confidence somatic alterations in 63% (20 of 32) of patients with CNS metastases of solid tumors, 50% (6 of 12) of patients with primary brain tumors, and 0% (0 of 9) of patients without CNS involvement by cancer [108]. Wang *et al*. found that all medulloblastomas, ependymomas, and high-grade gliomas that abutted a CSF space or cortical surface were detectable (100% of 21 cases; 95% CI = 88–100%), whereas no CSF tumor DNA was detected in patients whose tumors were not directly adjacent to a CSF reservoir [109].

In De Mattos-Arruda *et al*.'s seminal work, CSF ctDNA was identified in brain primary and metastatic tumors and represented private mutations from CNS lesions. Furthermore, sensitivity of ctDNA for somatic mutations of the CNS was higher than plasma DNA in patients with a CNS-restricted disease (58% *vs*. 0%, *p* = 0.0006). While in patients with the abundant visceral disease, the sensitivity of CNS DNA and plasma DNA was comparable (60.5% *vs*. 55.5%). The investigators further monitored the change of mutant allelic frequency (MAF) of CSF DNA and plasma DNA in a serial study. MAFs of CSF DNA decreased with surgical resection and/or responses to systemic therapy and increased with tumor progression [7]. Similar findings were also presented by Pan *et al*. The median concentration of cfDNA in CSF is lower than that in plasma (2.1 ng/mL *vs*. 7.7 ng/mL). However, the ability to detect mutations in CSF is stronger than in plasma in brain tumor patients with low systemic metastatic burden [19]. EGFR mutation in CSF was also detected in a case with suspected leptomeningeal metastasis from EGFR mutant lung adenocarcinoma, which indicates the characterization of brain tumor genomic aberrations through CSF DNA analysis is possible. Very few cells are present in CSF under routine conditions (0–5 cells/L). The scarcity of cells in CSF may reduce the background noise from normal DNA when detecting mutations [19].

## CONCLUSIONS

Non-blood circulating tumor DNA has an enormous potential for large-scale screening of local neoplasms because of its non-invasive nature, close proximity to the tumors, easiness and it is an economically viable option. It permits longitudinal assessments and allows sequential monitoring of response and progression [110]. The direct contact of cancerous cells and body fluid may facilitate the detection of tumor DNA, while vascular invasion likely happens at a later stage in tumorigenesis, which may explain the low sensitivity of plasma-based tests [111]. Furthermore, normal DNA always dilutes the ctDNA, which may be aggravated by inflammation and injury when very high amounts of normal DNA are released into the circulation [112]. Altogether, our review indicate that non-blood circulating tumor DNA presents an option where the disease can be tracked in a simple and less-invasive manner, allowing for serial sampling informing of the tumor heterogeneity and response to treatment.
